# “Every Woman Has a Different Cycle and Feels Differently”: A Qualitative Study of Athlete-Centred Perspectives on Menstrual Cycle Symptoms and Management in Female Endurance Sports

**DOI:** 10.3390/sports14050173

**Published:** 2026-04-24

**Authors:** Elena Liebrenz, Alexander Smith, Michael Liebrenz, Jill Colangelo, Ana Buadze

**Affiliations:** 1 Department of Psychiatry, Psychotherapy and Psychosomatics, Psychiatric Hospital, University of Zurich, 8091 Zurich, Switzerland; elena.liebrenz@icloud.com; 2 Department of Forensic Psychiatry, University of Bern, Hochulstrasse 4, 3012 Bern, Switzerland; 3 Faculty of Sport, Technology, and Health Sciences, St Mary’s University Twickenham, Twickenham TW1 4SX, UK

**Keywords:** menstrual cycle, endurance sport, cycle-based training, qualitative research, athlete wellbeing, self-regulation

## Abstract

Background: Although menstrual cycle-based training has attracted increasing attention in endurance sports, research has predominantly focused on ergometric parameters. However, the subjective perspectives and lived realities of athletes remain relatively underexamined. Accordingly, this study aimed to explore performance perceptions and self-regulatory experiences of female endurance athletes within real-life training and competitive contexts. Methods: Qualitative semi-structured interviews were conducted with twelve female endurance athletes (ages 18–42) across triathlon, running, swimming, cycling, and skiing. Data were analysed inductively using descriptive thematic analysis in MaxQDA. Results: Six themes emerged related to menstrual cycle experiences: body awareness and cycle-related perceptions; the influence of expectations and self-efficacy on perceived performance; heterogeneous approaches to cycle-based training; training and recovery adjustments; the ambivalent role of digital tracking tools; and communication openness and barriers. Overall, cycle-based training was applied inconsistently and served more as a framework for interpreting physical symptoms than as a means of optimising performance. Conclusions: In this sample of endurance athletes, cycle-related effects on performance and symptom perceptions were primarily shaped by biopsychosocial factors rather than physiological considerations alone. The menstrual cycle supported self-regulation, but rigid interpretations may risk reinforcing negative expectancies. These insights extend existing work by foregrounding athlete-centred, flexible approaches over deterministic training models.

## 1. Introduction

Sports science and sports medicine have historically been shaped by a male-dominated research paradigm directed towards the psychological and physiological needs of this demographic. As such, contemporary training models and performance benchmarks were predicated on samples primarily (or exclusively) consisting of male athletes, with females typically representing ≈35–44% of participants [1,2,3]. This entrenched gender imbalance means that female athlete-specific physiological and psychological characteristics, particularly those centred around the management of training and competition protocols at various stages of the menstrual cycle, have often been overlooked.

Hormonal fluctuations associated with the menstrual cycle have frequently been neglected, in part due to logistical, economic, and even biomechanical considerations [4]. Resultantly, female athletes have long been left to navigate adverse cycle-related symptoms in training and competition without optimal guidance and support. Separately, questions remain about whether training and nutrition protocols could be enhanced to work in concert with hormonal changes. Only in recent years has the international research community begun to close these gaps with targeted guidelines and female-inclusive study designs [5,6].

Specifically, the menstrual cycle may influence training and competitive outcomes, and this relationship may be bidirectional [7,8]. The menstrual cycle comprises the menstrual phase, the follicular phase, ovulation, and the luteal phase, wherein shifts in oestrogen and progesterone can affect cardiovascular, neuromuscular, metabolic, psychological, and thermoregulatory functioning [9,10,11,12,13]. Women’s personal experiences of cycle-related symptoms are wide-ranging and heterogeneous (e.g., fatigue around ovulation and cramping in the menstrual phase), which could impinge upon athletic performance, though objective performance indicators are not always impacted [14].

Some measurable outcomes remain debated in terms of their performance relevance; for example, oestradiol may promote lipid oxidation when concentrations are elevated (i.e., in the late follicular and luteal phases), potentially sparing glycogen reserves during endurance exercise [15]. Furthermore, progesterone in the luteal phase increases resting energy expenditure and basal body temperature and could impair thermoregulation under conditions of intense activity [12,13]. Neuromuscular function can also vary, with some studies suggesting marginally higher muscular strength around ovulation, but meta-analytic evidence indicates that these effects are generally negligible and methodology-dependent; greater subjective fatigue has been more consistently reported in the luteal phase, whereas objective recovery markers do not reliably differ between phases [9,16,17].

These variations suggest that physical capacity, perceived exertion, and recovery could differ substantively across the cycle. Such dynamics may be apparent in endurance sports, where sustained aerobic output places continuous demands on metabolic and thermoregulatory systems [16]. Equally, the high training loads and potential for low energy availability (LEA) in endurance sports may induce menstrual disturbances, thereby influencing symptom severity (e.g., for dysmenorrhoea, oligomenorrhoea, and exercise-associated amenorrhoea) [7,8]. Additionally, progesterone metabolites modulate GABAergic systems in the central nervous system, which in turn can contribute to the mood shifts and concentration difficulties identified in the late luteal phase, particularly for those with premenstrual syndrome or premenstrual dysphoric disorder [18,19].

Empirical data on performance remains equivocal and only partially generalisable. Although some studies highlight diminished performance, especially in the early follicular phase, others find no significant effects [20]. This is further complicated by methodological limitations, including variations in phase recognition, variations in hormonal contraception use, and distinct outcome measures, as well as terminological ambiguities [5,20,21]. Indeed, terms like “cycle-based”, “cycle-oriented”, or “cycle-synced” training have been adopted interchangeably to describe methods wherein training parameters are adjusted according to menstrual cycle phases [5,22]. Given the lack of standardised nomenclature, “cycle-based training” is hereafter employed as an umbrella term to encompass any approach that tailors training to menstrual cycle phases, in line with prevailing usage in the sports science literature. Current evidence may not adequately address the differences between providing guidance based on performance-based agendas and athlete concerns about symptom management [5,23]. Ultimately, these inconsistencies hamper cross-comparability and engender additional practical uncertainties.

Despite this, cycle-based training has recently gained considerable visibility in coaching and training planning. Social media influencers, athlete blogs, and commercialised programmes have increasingly begun to promote cycle-based adjustments to training, nutrition, and recovery, which at times have been devoid of scientific validation (e.g., [23,24]). Yet, this trend has not gone unchallenged, with recent media commentary questioning cycle-based training and warning that generic recommendations may lead women to undertrain, avoid intensity without physiological justification, or disregard personal agency surrounding management strategies [24,25].

Existing scholarship has highlighted a lack of knowledge among coaches and suggested that the need for training alterations to achieve performance goals may be overstated, confusing, and engender increased stigma [26,27]. Consequently, researchers have cautioned against blanket endorsements for this type of training, instead accentuating a need for context-sensitive approaches to symptom management [23]. In light of this, a disconnect endures between the growing interest in cycle-based training and menstrual cycle symptom management, as well as the limited understanding of how athletes might experience and apply solutions [28].

While ergometric measurements can provide insights into optimal training plans, individualised cycle-related experiences (e.g., body awareness, self-efficacy, and subjective performance perceptions) themselves constitute critical data that quantitative approaches alone cannot capture. Qualitative insights are therefore needed to move beyond physiological findings and explore how female athletes interpret cycle-related changes within real-life training and competitive environments. Hence, through semi-structured interviews, this study sought to foreground athlete-centred perspectives on menstrual cycle management and its perceived relevance in endurance sports.

## 2. Methods

### 2.1. Study Design

A descriptive, qualitative design was employed to explore attitudes, perceptions, and experiences among female endurance athletes regarding their menstrual cycle in sporting contexts.

### 2.2. Sample and Recruitment

Inclusion was limited to female endurance athletes aged 18 or older who trained at least five hours per week across sports including triathlon, running, swimming, cycling, and skiing. Endurance athletes were selected due to their reliance on sustained physiological output, where cycle-related fluctuations in energy metabolism, thermoregulation, and recovery may be particularly salient [21,22,23].

With this in mind, interviewees were identified through purposive and snowball sampling, which may have influenced the composition of the sample. Specifically, initial recruitment occurred via the first author’s sporting networks; additional participants were contacted via targeted outreach on Instagram, including direct exchanges with athletes followed by national sporting organisations, a recruitment post shared by a prominent endurance athlete with a substantial follower base, and circulation of a digital flyer.

This yielded a sample of twelve female endurance athletes (aged 18–42; *M* = 27.8 years) representing various disciplines, encompassing triathlon (*n* = 5), swimming (*n* = 2), road cycling (*n* = 2), marathon running (*n* = 1), mountain biking (*n* = 1), and skiing (*n* = 1). Six participants trained at elite or national squad level and six were competitive amateurs. Half of the athletes reported actively incorporating cycle-based approaches into their training regimens.

Menstrual cycle characteristics (e.g., regularity, symptoms) and contraceptive use varied across the sample, as did training volume. Most participants had regular menstrual cycles, although some noted irregularities or previous amenorrhea linked to high training loads; contraceptive use also varied between interviewees. Weekly training volume ranged from 6 to 25 h (*M* = 17.4). These variables were not controlled analytically given the study’s aim of exploring subjective experiences as opposed to comparing predefined subgroups.

### 2.3. Data Collection

Data were collected via semi-structured interviews conducted online by the first author between February and September 2025. Each interview lasted approximately 60–90 min, was audio-recorded with participant consent, and thereafter transcribed verbatim; all transcripts were anonymised prior to data analysis.

A topic guide was employed comprising seven thematic areas: (1) sporting biography, demographic questions, and training background; (2) understanding and definition of cycle-based training; (3) implementation and specific adjustments; (4) subjective effects and body awareness; (5) performance development and indicators; (6) challenges and barriers; and (7) recommendations and open reflections. The topic guide was developed in consultation with experienced qualitative researchers in the authorship team (AB and ML) and was informally tested during the first three interviews and iteratively refined to improve clarity, relevance, and flow; minor adjustments were made as data collection progressed in response to participant feedback and emerging thematic insights. The semi-structured design allowed for flexibility and responsiveness during interviews, with scope for interviewees to elaborate on their experiences and introduce topics.

Throughout the interviews, the sequence of questions was adapted flexibly to facilitate natural conversational flow. Follow-up probes were used to clarify personal perceptions and elicit further details, while open-ended questions and neutral prompts minimised interviewer-driven influences and convergences, allowing participants to guide the discussion and articulate diverse perspectives. Moreover, in the data collection period, the first author maintained brief memos and observational notes to capture impressions (e.g., non-verbal cues and emotional responses). These notes informed the subsequent analysis and contextualisation of the interviewee responses.

### 2.4. Data Analysis

Data analysis was conducted within an interpretivist framework, assuming that participants’ experiences were subjectively constructed and context-dependent. Transcripts underwent descriptive thematic analysis, led by the first author and supported by MaxQDA software (Version 2024). Following multiple close readings to ensure familiarity with the data, relevant passages were assigned descriptive codes (e.g., “fatigue before menstruation”, “increased energy in first phase”, “coach uncertainty”), reflecting an initial open coding process. Subsequently, in a second step, related codes were grouped into higher-order categories and iteratively refined into six overarching final themes.

Codes and themes were continuously reviewed and consolidated during the analytic process. Specifically, the selection and weighting of themes was guided by their salience within the data. To enhance analytical rigour, emerging codes and themes were regularly discussed within the research team, and discrepancies were addressed through reflexive dialogue and iterative comparisons.

Thematic saturation was operationalised as the point at which no new codes, categories, or conceptual insights could be discerned. This was observed after approximately ten interviews, with the final two confirming previously identified patterns, thus supporting the adequacy of the sample size for the exploratory study objectives.

### 2.5. Ethical Considerations

As this investigation addressed health-related topics, a clarification of responsibility was submitted to the Cantonal Ethics Committee of Bern, which issued a declaration of non-jurisdiction under the Swiss Human Research Act (Req-2025-00644), indicating that formal ethical approval was not required [29]. Nevertheless, the study upheld ethical best practices in line with the Declaration of Helsinki [30]. Prior to each interview, participants received an informed consent form outlining the study’s purpose, procedures, and their rights; consent was provided electronically, including for audio recording. All data were stored securely, with identifying information removed and participant codes used throughout (i.e., P01–12).

## 3. Results

### 3.1. Overview of Themes

The descriptive thematic analysis yielded six overarching themes reflecting how female endurance athletes perceive their training and performance within the context of menstrual cycle-related changes: (1) performance-related body awareness across the menstrual cycle; (2) the influence of expectations and self-efficacy on perceived performance; (3) heterogeneous approaches to cycle-based training; (4) training and recovery adjustments; (5) ambivalent effects of digital tracking tools; and (6) openness and barriers in communication. Table 1 outlines these themes supported by illustrative quotations, and each theme is outlined in further detail in the ensuing subsections.

Although closely related, these themes embody distinct aspects of athletes’ experiences. For example, “Body awareness” refers to the perception of physical sensations across the menstrual cycle, whereas “performance perception” reflects how these sensations are interpreted in relation to expected and actual performance. In turn, “Self-efficacy” captures interviewees’ confidence in their ability to perform despite cycle-related fluctuations.

### 3.2. Body Awareness and Cycle-Related Perceptions

As highlighted in Table 1, a central theme concerned how participants perceived bodily sensations and performance readiness across different points in the menstrual cycle, and how these perceptions informed their understanding of cycle-related changes in training and competition. Specifically, the interviewees discussed a range of physical symptoms and sensations, from increased energy and lightness to fatigue and heaviness, with variation across the sample.

Many athletes depicted a sense of reduced capacity in the premenstrual and early menstrual phases. P12 (road cyclist) attested: “I think my weakest phase is always the one before menstruation […] sometimes there are days on the bike where you just have no power in your legs”. Similarly, P09 (swimmer) observed: “Maybe a week to half a week before my period, I feel more tired, a bit weaker”. P10 (road cyclist) described feeling “a bit like a hippopotamus—very sluggish, everything is slower and more effortful than usual”.

In contrast, several interviewees noted heightened energy and better performance outcomes in the follicular phase or immediately following menstruation. P12 felt “strongest directly after menstruation”, while P09 experienced a “hero feeling” during her cycle, asserting that “I always have this feeling that I’m a hero because I can do whatever I want at that time”. Equally, P08 (triathlete) reported: “I would say I feel strong when I have my period—before, that wasn’t the case”.

However, the relationship between subjective perceptions of performance readiness and objective indicators was not always consistent. P03 (mountain biker) reflected: “Actually, while I have my period, I don’t feel that great, but I don’t think my performance suffers. I’ve had my best races while on my period”.

Collectively, these accounts suggest that the menstrual cycle was experienced primarily through subjective bodily awareness, but that these perceptions did not consistently manifest as perceived or actual performance decrements.

### 3.3. Influence of Expectations and Self-Efficacy on Perceived Performance

Psychological factors shaped how athletes perceived, interpreted, and responded to the effects of cycle-related symptoms on training and competition (Table 1).

Several interviewees identified how anticipating poor performance could become self-fulfilling; for example, P04 (cyclist/skier) stated: “I can imagine that you talk yourself into it—‘Oh, this won’t work anyway’—or have a mental breakdown”. This athlete further asserted that awareness of being in a so-called “weaker phase” could detrimentally affect one’s mindset before training commences. In this sense, menstrual cycle awareness was not neutral; for this interviewee, merely identifying a timepoint as a “bad” or “weak” phase appeared to influence confidence and readiness prior to exercise.

Conversely, athletes with higher self-efficacy appeared better able to maintain performance, even with cycle-related discomfort. P02 (marathon runner) described developing a sense of mental resilience: “I’ve trained myself to have the self-confidence to say: I try to influence everything I can on race day. And what I can’t influence—like the weather, like my cycle—I completely block out, because there’s no point having negative thoughts about things I can’t change”.

Additionally, P03 (mountain biker) underlined this capacity to dissociate subjective feelings from performance outcomes, recalling strong competitive performances despite self-reported physiological limitations. These insights indicate that the menstrual cycle influenced performance perceptions through physical symptoms and also via expectation-related processes, self-efficacy, and the meaning athletes attached to specific phases.

### 3.4. Heterogeneous Approaches Towards Cycle-Based Training

Participants varied considerably in how they understood and operationalised menstrual cycle-informed training, with no consistent approach emerging (Table 1); some followed structured, phase-specific programmes, whereas others exhibited only a vague awareness of the concept or even dismissed its relevance entirely.

Certain athletes followed explicit cycle-informed protocols. P04 (cyclist/skier) explained: “I have the plan of my cycle and I base my training on that […] sometimes I adjust it a bit depending on how I feel or how much time I have”. P10 (road cyclist) delineated systematic strategies: “Training is adapted to cycle phases: in the first half more intensive with intervals and strength training, in the second half more base work and less intensive sessions”.

Others adopted a more intuitive approach without direct reference to cycle phases, adjusting based on day-to-day sensations in ways that were not always explicitly distinguished from general training responsiveness. P03 (mountain biker) reflected: “I know when I have my period and if I have it, I don’t do intervals, but then I see day by day how I feel and adjust the training if I’m not feeling good”.

Separately, certain interviewees expressed uncertainty about what cycle-based training entailed and its purported effects. Notably, P07 (triathlete) conceded: “I’ve never really looked into cycle-based training in depth, even though I’ve heard of it”. P05 (triathlete) reported noticing little difference across her cycle and rarely considered whether her sensations might be cycle-related. Overall, the interviews showed that menstrual cycle relevance varied; for some athletes it explicitly informed training plans, whereas for others it remained peripheral and uncertain.

### 3.5. Training and Recovery Adjustments

When participants described adjustments to training or recovery, these were commonly framed as responses to menstrual cycle-related symptoms as opposed to adherence to rigid phase-based programmes (Table 1). These changes were typically guided by how athletes felt at particular menstrual cycle timepoints (especially premenstrually or during menstruation), although some interviewees also acknowledged that these modifications overlapped with day-to-day awareness of fatigue and recovery needs.

A reduction in training load was a commonly adopted approach. P02 (marathon runner) explained: “I always try to maintain the kilometres, but I really vary the pace targets”. P01 (swimmer) asserted: “Where I had my period, we scaled back a bit on intensity”; P10 (road cyclist) similarly adjusted her training load across phases.

Some participants altered the type of training instead of simply moderating volume or intensity. For example, P04 (cyclist/skier) described switching to gentler activities: “In the fourth week, I find zone 2 inline skating great because it doesn’t stress the musculoskeletal system as much as running, where you’re constantly jumping on your joints”.

Nutritional changes were also reported. P08 (triathlete) attested: “I eat more carbohydrates or sweets maybe before the period, and then I have no appetite”. Athletes who made these adjustments reported various benefits, including improved body awareness and reduced illness. P04 stated: “Since then, I’m much less sick. Before, I was constantly catching colds”, and P10 echoed this: “I definitely feel less sick, which I attribute to less training at the limit and better recovery and nutrition”. Hence, the menstrual cycle appeared to shape training and recovery mainly by prompting flexible symptom management as opposed to serving as a standardised performance optimisation model.

### 3.6. Ambivalent Effects of Digital Tracking Tools

Digital tools were commonly employed to monitor menstrual cycle timing and associated physiological signals, but participants diverged in whether such tracking clarified or complicated their understanding of cycle-related effects on training and performance.

Many interviewees used tools such as Garmin, Flo, Whoop, or Oura Ring to monitor cycle phases, heart rate variability (HRV), sleep quality, and additional parameters. Specifically, P04 (cyclist/skier) described how HRV data prompted her awareness of cycle effects: “It was actually through the HRV, seeing that curve, that I first realised the cycle really does have an impact on the body”.

For some, tracking enhanced self-understanding and supported training decisions. P01 (swimmer) maintained: “I feel like I pay more attention to why I feel the way I do—why I feel powerful, when I feel low on energy”. P11 (skier) reported using daily readiness measurements: “I do a measurement every morning to see how my body is doing, and it shows when I’m not ready to train fully”. For this participant, tracking made menstrual cycle patterns more visible and helped support the interpretation of symptoms, readiness, or recovery in cycle-related terms.

Nevertheless, other athletes deemed these forms of tracking to be confusing and even unhelpful. P05 (triathlete) observed: “I use Whoop and see which phase I’m in; it says how you should typically feel. I find that interesting—‘this is how I should feel now’—but I don’t really notice a big difference”.

For some participants, this disconnect between expected and actual symptoms could generate uncertainty; several expressed frustration at the lack of scientific guidance for interpreting their data. P04 stated: “When I noticed the HRV thing, I tried to find information about whether it’s really like that during the cycle, and I found nothing—not a single study or anything”. Accordingly, digital tracking appeared to make the menstrual cycle more salient in training, but this was deemed either beneficial or burdensome depending on whether the data aligned with lived experiences.

### 3.7. Openness and Barriers in Communication

Experiences of discussing the menstrual cycle within sporting environments differed (Table 1). Certain participants described supportive cultures, whereas other interviewees encountered silence and discomfort surrounding the topic.

Some athletes reported positive team environments and open discussions. P11 (skier) recounted: “We once just talked about it in the team—who does what—and with us, almost everyone does something similar”. This respondent suggested that this informal peer exchange facilitated knowledge-sharing and normalisation.

A number of interviewees received adequate support from coaches. For example, P01 (swimmer) recalled a coach’s response when wanting to adjust period timing for a competition: “He said no, if I’ve never done it, don’t—we’ll just train more specifically, with energy levels and so on”. P10 (road cyclist) depicted an established communication system: “I always make a note on the first day of a new cycle in TrainingPeaks […] if there are any other issues, I add a comment under the training and then we can adjust”. Separately, P10 praised the proactivity towards this subject: “I have the privilege of being in a national team where this topic was handled extremely well, through presentations and personal contacts”.

However, others identified significant gaps. P09 (swimmer) stated bluntly: “I believe that education by coaches regarding the female cycle in swimming is insufficient”, contrasting this with athletics, where more awareness was discerned. P05 (triathlete) noted: “I haven’t seen a coach who really offers this and adjusts accordingly. I understand why. It’s very individual”.

Structural barriers to discussing or acting on menstrual cycle-related needs were also apparent. P11 (skier) underlined the impracticality of individualised cycle-based planning in team settings: “In winter, we can’t really adjust it […] we have our training plans and everyone in the team has different cycles, so you can’t adapt it”.

Fear of being perceived as weak or making excuses appeared to inhibit some athletes from raising the topic. P02 (marathon runner) reflected that men “have no idea” what women experience, highlighting a need for greater sensitivity. P10 (road cyclist) attested that elite sport is highly result-oriented, with many unwilling to sacrifice short-term performance for longer-term wellbeing.

These accounts suggest that the menstrual cycle influenced sport both through symptoms themselves and through athletes’ capacity to communicate those experiences and have them taken seriously within performance-oriented settings.

## 4. Discussion

This qualitative study explored how female endurance athletes perceive and manage their menstrual cycle and related symptoms in real-life training and competitive scenarios, alongside attitudes towards cycle-based training approaches. In doing so, it brings nuanced, athlete-centred insights into an area that has been shaped largely by quantitative debates and historical gendered gaps [1,2,3,19,20]. Collectively, these findings underline that cycle-related shifts are not purely physiological and appeared to involve biopsychosocial influences; participants depicted bodily symptoms (e.g., fatigue, heaviness, gastrointestinal discomfort, and pain) together with psychological processes (e.g., confidence, self-efficacy, and anticipatory beliefs), all integrated within broader sporting environments conditioned by coaching norms and team cultures. Attempts to incorporate digital tracking tools represented a further mechanism through which athletes sought to contend with these influences.

This reinforces previous qualitative data, which similarly highlighted a compound interplay involving physiological, psychological, and social components for understanding how athletes navigate menstrual cycle-related symptoms [31]. Interestingly, numerous participants in the current study reported feeling physically suboptimal at certain points in their cycle yet still attained strong results, indicating that cycle-related experiences were highly individualised and rarely reducible to a single “phase effect”. Other samples of endurance athletes perceived menstruation as detrimentally affecting performance, highlighting the difficulties of translating physiological plausibility into reliable cycle-based changes [20,32,33].

The mismatch between subjective states and objective outcomes observed in this sample may well reflect pertinent dynamics beyond endocrine physiology, thus extending prior quantitative findings. Prospective research found that variations in performance correlate more strongly with self-described psychological wellbeing than with fluctuations in sex hormones, notwithstanding some phase-associated decrements [34]. Cognitive testing has revealed that personal perceptions of impairment during menstruation were incongruent with objective performance, which was paradoxically superior during menstruation relative to participants’ symptom reports and expectations [35,36].

In the endurance disciplines captured in this study, where pain tolerance can constitute a performance determinant and a form of cultural currency (e.g., [37,38]), menstrual cycle-related symptoms may alter the perception of readiness, motivation, and recovery. Indeed, in this sample, cycle-based training was foregrounded more as a form of personal interpretation than as a concerted performance optimisation strategy. Yet, for some interviewees, awareness of being in a so-called “weaker phase” adversely affected their pre-training mindset, implying a plausible nocebo pathway, wherein cycle-based frameworks may conceivably reinforce negative expectancies if overemphasised, though this cannot be fully confirmed by the current data [39]. Importantly, adverse perceptions of symptoms and complications during the menstrual cycle could hinder efforts to avoid LEA and ensuing hypothalamic amenorrhea.

Consequently, these insights potentially undermine increasingly commercialised messaging surrounding cycle-based training, which frequently imply guaranteed benefits [23,24,25]. Instead, cycle-based training emerged as a nebulous and unevenly applied concept during the interviews. Practices ranged from structured cycle-based periodisation to intuitive daily shifts based on symptoms, with some athletes dismissing them entirely when discerning little difference across their cycle; this aligns with cautions in the literature against blanket cycle-based directives [20]. Ultimately, it remains unclear whether future research should continue to advocate for comprehensive guidance, especially where private industry-driven research may skew both the need for and the value of such recommendations. More broadly, this exemplifies the contested landscape in this domain; even when physiological rationales exist, robust scientific support for cycle-based programming has not been comprehensively established [6,23].

Separately, digital tools were widely utilised among this sample but the relationship between tracking and self-understanding was not straightforward. These technologies were simultaneously portrayed as an empowering but burdensome feature of menstrual cycle management. Whether tracking influenced training decisions, confidence, or symptom interpretation in consistent ways was indiscernible from these interviews. Responses were mixed and appeared to depend largely on the degree to which app-generated information corresponded with actual physical sensations, suggesting that individual variation in this domain warrants dedicated investigation.

This pattern of ambivalence mirrors the wider literature in the context of menstrual cycles. Users can value prediction and preparation, but discrepancies and algorithmic standards can induce anxiety and confusion [40]. Notably, in an appraisal of iPhone fertility and menstrual tracking apps, ≈20% contained serious inaccuracies, and overall quality was modest [41]. As some interviewees attested, app-generated menstrual cycle narratives risk undermining autonomy and amplifying detrimental expectancy effects, particularly if training decisions are predicated on data that do not adequately reflect lived experiences.

Moreover, discussions about the menstrual cycle in sporting settings diverged considerably between interviewees, ranging from open dialogues to reluctance and avoidance. The spectrum in this sample resonates with prior work identifying communication issues between female athletes and coaching staff, with the present data exploring athletes’ capacity to interpret and act on menstrual cycle-related experiences in their sporting environments [31,42].

## 5. Strengths, Limitations, and Future Research Directions

This study yielded qualitative insights into how female endurance athletes subjectively experience and manage their menstrual cycle in training and competitive contexts; this area remains underexplored in the existing literature, which has largely focused on quantifiable, ergometric data. Notably, the diverse range of participants bolstered the breadth of perspectives in the results, spanning multiple endurance disciplines, competitive levels, and demographics. Moreover, the attainment of thematic saturation suggests there was sufficient depth to underpin the exploratory research objectives.

Nevertheless, there were several limitations in the study design. Although saturation was observed and the sample size aligned with other qualitative studies on endurance athletes, transferability to other populations or sporting frameworks cannot therefore be assumed [33,41,43]. Recruitment via sporting networks and social media outreach may have led to selection bias, potentially resulting in a sample with greater awareness of or engagement with cycle-based training. This enabled the recruitment of athletes with relevant experience, but may mean that perspectives from those less engaged with the topic are underrepresented, thus impinging on the generalisability of the results. Relatedly, the sample consisted exclusively of athletes willing to discuss menstrual cycle experiences in an interview setting, which may have narrowed the scope of the findings.

Additionally, as an interview-based investigation, menstrual cycle phase was not independently verified and the accounts from participants were derived from subjective self-report and retrospective self-tracking, which could have introduced recall bias. Interview framing around the menstrual cycle may have encouraged some participants to attribute training adjustments to cycle-related causes that were in practice more general responses to perceived readiness or fatigue.

The lack of formal pilot testing of the topic guide a priori may have limited opportunities for granular refinement of question phrasing and sequencing before data collection. Similarly, social desirability could have affected participant answers about training adjustments and performance perceptions. Equally, the position of the first author as an active endurance athlete facilitated rapport, but may also have entailed potential biases [44]; to mitigate this, reflexive memos were maintained, as were regular consultations with other members of the research team.

Notwithstanding these limitations, the findings hold implications for athletes, coaches, practitioners, and other sports-adjacent stakeholders, providing important future research foundations. For example, longitudinal and mixed-method designs would help elucidate how experiences of the menstrual cycle develop over time and clarify the relationship between perceived symptoms and objective physiological markers. Likewise, expanding the scope of inquiry to encompass team sports, distinctive cultural settings, and athletes using hormonal contraception would strengthen generalisability.

Given the ambivalent attitudes towards digital tracking in this sample and elsewhere, examining how athletes interpret cycle-related data, and how such tools might better support (rather than overwhelm), is a pertinent issue for further investigation. The present qualitative data suggest that work in this area should extend beyond physiological measurements to incorporate psychological considerations; these could include expectancy pathways (e.g., how app-generated phase narratives shape confidence, effort, and self-talk), self-efficacy, and intuition, since nocebo mechanisms may affect performance [45].

## 6. Conclusions

Using a qualitative design, this study sought to explore perspectives on cycle-based training and its perceived relevance in endurance sports, thereby foregrounding athlete-centred insights within this contested area. Collectively, the results from this sample highlight that menstrual cycle experiences were individualised and cannot be meaningfully reduced to uniform cycle-based patterns and generalised training prescriptions. Notably, perceptions of cycle-related symptoms were context-dependent and shaped by an interplay of physiological sensations, psychological processes, and the social environments in which athletes train and compete. Hence, rather than primarily constituting a tool for performance optimisation, awareness of menstrual cycles appeared to serve as a form of personal interpretation and self-regulation for these athletes; this entailed possible benefits but also broader risks if linked to rigid expectations and commercially driven narratives.

Moreover, these findings suggest that some athletes favoured flexible, athlete-centred approaches over deterministic cycle-based models. The interviews indicate that positioning menstrual health as a routine and contextual component of athlete care may help support both performance sustainability and the wellbeing of female athletes. Ultimately, as one participant succinctly observed: “every woman has a different cycle and feels differently”.

## Figures and Tables

**Table 1:** Overview of themes and example quotes.

Theme	Description	Example Participant Quote *
(1) Body awareness and cycle-related perceptions	Interviewees reported fluctuations in energy, fatigue, and physical sensations across menstrual cycle phases, with considerable inter-individual variation.	“You feel a bit like a hippopotamus—very sluggish, everything is slower and more effortful than usual” (P10).
(2) Influence of expectations and self-efficacy on perceived performance	Anticipatory beliefs and mindset shaped how participants interpreted and responded to cycle-related sensations.	“I’ve trained myself to have the self-confidence to say: what I can’t influence, like the weather, like my cycle, I completely block out” (P02).
(3) Heterogeneous approaches towards cycle-based training	Understanding and implementation of cycle-based training varied widely, from structured plans to intuitive adjustments.	“I know when I have my period and if I have it, I don’t do intervals, but then I see day by day how I feel” (P03).
(4) Training and recovery adjustments	Participants described phase-specific modifications to intensity, volume, and recovery strategies.	“Since then, I’m much less sick. Before, I was constantly catching colds.” (P04).
(5) Ambivalent effects of digital tracking tools	Apps and wearables supported self-understanding but could also engender pressure and confusion.	“It was actually through the HRV (heart rate variability), seeing that curve, that I first realised the cycle really does have an impact on the body” (P04).
(6) Openness and barriers in communication	Communication about the cycle with coaches and teammates ranged from open dialogue to reluctance and uncertainty.	“We once just talked about it in the team—who does what—and with us, almost everyone does something similar” (P11).

* *Note*. PX indicates participant code.

## Data Availability

The original contributions presented in this study are included in the article. Further inquiries can be directed to the corresponding authors.

## References

[1] Costello J.T., Bieuzen F., Bleakley C.M. (2014). Where are all the female participants in Sports and Exercise Medicine research?. Eur. J. Sport. Sci..

[2] Ose B.M., Eisenhauer J., Roepe I., Herda A.A., Vopat B.G., Vopat L.M. (2025). Where Are All the Female Participants in Sports and Exercise Medicine Research? A Decade Later. Am. J. Sports Med..

[3] Cowley E.S., Moore S.R., Olenick A.A., McNulty K.L. (2021). “Invisible Sportswomen”: The Sex Data Gap in Sport and Exercise Science Research. Women Sport Phys. Act. J..

[4] Emmonds S., Heyward O., Jones B. (2019). The Challenge of Applying and Undertaking Research in Female Sport. Sports Med. Open..

[5] Elliott-Sale K.J., Minahan C.L., de Jonge X.A.K.J., Ackerman K.E., Sipilä S., Constantini N.W., Hackney A.C. (2021). Methodological Considerations for Studies in Sport and Exercise Science with Women as Participants: A Working Guide for Standards of Practice for Research on Women. Sports Med..

[6] Bruinvels G., Burden R.J., McGregor A.J., Ackerman K.E., Dishi M., Lewis N.A., Pedlar C.R. (2021). Sport, Exercise, and the Menstrual Cycle: Where Is the Research?. Br. J. Sports Med..

[7] Mountjoy M., Ackerman K.E., Bailey D.M., Burke L.M., Constantini N., Hackney A.C., Heikura I.A., Melin A., Pensgaard A.M., Stellingwerff T. (2023). 2023 International Olympic Committee’s (IOC) Consensus Statement on Relative Energy Deficiency in Sport (REDs). Br. J. Sports Med..

[8] Colangelo J., Smith A., Henninger K., Buadze A., Liebrenz M. (2025). Exploring the Presentation of REDs in Ultra Endurance Sport: A Review. J. Eat. Disord..

[9] Reed B.G., Carr B.R., Feingold K.R. (2018). The Normal Menstrual Cycle and the Control of Ovulation. Endotext.

[10] Thiyagarajan D.K., Basit H., Jeanmonod R. (2024). Physiology, Menstrual Cycle. StatPearls.

[11] Baker F.C., Siboza F., Fuller A. (2020). Temperature Regulation in Women: Effects of the Menstrual Cycle. Temperature.

[12] Benton M.J., Hutchins A.M., Dawes J.J. (2020). Effect of Menstrual Cycle on Resting Metabolism: A Systematic Review and Meta-Analysis. PLoS ONE.

[13] Yildirir A., Kabakci G., Akgul E., Tokgozoglu L., Oto A. (2001). Effects of Menstrual Cycle on Cardiac Autonomic Innervation as Assessed by Heart Rate Variability. Ann. Noninvasive Electrocardiol..

[14] Kishali N.F., Imamoglu O., Katkat D., Atan T., Akyol P. (2006). Effects of menstrual cycle on sports performance. Int. J. Neurosci..

[15] Willett H.N., Hackney A.C., Etnier J.L. (2021). Influence of Menstrual Cycle Estradiol-β-17 Fluctuations on Energy Substrate Utilization-Oxidation during Aerobic, Endurance Exercise. Int. J. Environ. Res. Public Health.

[16] Carmichael M.A., Thomson R.L., Moran L.J., Wycherley T.P. (2021). The Impact of Menstrual Cycle Phase on Athletes’ Performance: A Narrative Review. Int. J. Environ. Res. Public Health.

[17] Taylor M.Y., Hrozanova M., Nordengen L., Sandbakk Ø., Osborne J.O., Noordhof D.A. (2024). Influence of Menstrual-Cycle Phase on Sleep and Recovery Following High- and Low-Intensity Training in Eumenorrheic Endurance-Trained Women: The Female Endurance Athlete Project. Int. J. Sports Physiol. Perform..

[18] Paludo A.C., Paravlic A., Dvořáková K., Gimunová M. (2022). The Effect of Menstrual Cycle on Perceptual Responses in Athletes: A Systematic Review with Meta-Analysis. Front. Psychol..

[19] Hantsoo L., Epperson C.N. (2020). Allopregnanolone in premenstrual dysphoric disorder (PMDD): Evidence for dysregulated sensitivity to GABA-A receptor modulating neuroactive steroids across the menstrual cycle. Neurobiol. Stress.

[20] McNulty K.L., Elliott-Sale K.J., Dolan E., Swinton P.A., Ansdell P., Goodall S., Thomas K., Hicks K.M. (2020). The Effects of Menstrual Cycle Phase on Exercise Performance in Eumenorrheic Women: A Systematic Review and Meta-Analysis. Sports Med..

[21] Elorduy-Terrado A., Torres-Luque G., Radesca K., Muñoz-Andradas G., Saenz-Bravo M., Domínguez-Balmaseda D. (2025). Evaluation the Impact of Hormonal Fluctuations During the Menstrual Cycle on the Performance of Female Athletes-Systematic Review. Muscles.

[22] Meignié A., Duclos M., Carling C., Orhant E., Provost P., Toussaint J.F., Antero J. (2021). The Effects of Menstrual Cycle Phase on Elite Athlete Performance: A Critical and Systematic Review. Front. Physiol..

[23] Mikkonen R.S., Häkkinen K. (2025). Evidence for Periodizing Strength and/or Endurance Training According to Menstrual Cycle Phases to Optimize Female Athlete Performance Is Lacking. Strength Cond. J..

[24] Wilkins B. Controversial Studies Suggest Cycle Syncing Is a Scam—Here’s How It Could Be Detrimental. *Women’s Health UK* 2025. https://www.womenshealthmag.com/uk/fitness/.

[25] CrossFit Kreis 9 (2025). Cycle Syncing Workouts: Why This Fitness Trend Is Holding Women Back. https://www.crossfitkreis9.ch/cycle-syncing-workouts/.

[26] Colenso-Semple L.M., D’Souza A.C., Elliott-Sale K.J., Phillips S.M. (2023). Current evidence shows no influence of women’s menstrual cycle phase on acute strength performance or adaptations to resistance exercise training. Front. Sports Act. Living.

[27] Steel K.A., O’Shea M., Nelson M. (2025). An Intersectional Approach: Carving Out a Contemporary Research and Practice Agenda (with and for) Female Athletes. Int. J. Sports Sci. Coach..

[28] van den Berg C.A., Doyle-Baker P.K. (2025). Are we asking the right questions? Female athletes’ perspectives on the menstrual cycle in sport. Psychol. Sport Exerc..

[29] Fedlex (2023). Swiss Federal Act on Research Involving Human Beings. https://www.fedlex.admin.ch/eli/cc/2013/617/en.

[30] World Medical Association (2024). WMA Declaration of Helsinki—Ethical Principles for Medical Research Involving Human Participants. https://www.wma.net/policies-post/wma-declaration-of-helsinki/.

[31] Findlay R.J., Macrae E.H.R., Whyte I.Y., Easton C., Forrest Née Whyte L.J. (2020). How the Menstrual Cycle and Menstruation Affect Sporting Performance: Experiences and Perceptions of Elite Female Rugby Players. Br. J. Sports Med..

[32] Oosthuyse T., Bosch A.N. (2010). The Effect of the Menstrual Cycle on Exercise Metabolism: Implications for Exercise Performance in Eumenorrhoeic Women. Sports Med..

[33] Günay A., Karahanoğlu A. (2026). Tracking Distances and Periods: Examining the Interplay between Endurance Sports and the Menstrual Cycle. Interact. Comput..

[34] Dam T.V., Dalgaard L.B., Sevdalis V., Bibby B.M., Janse de Jonge X., Gravholt C.H., Hansen M. (2022). Muscle Performance during the Menstrual Cycle Correlates with Psychological Well-Being, but Not Fluctuations in Sex Hormones. Med. Sci. Sports Exerc..

[35] Ronca F., Blodgett J.M., Bruinvels G., Lowery M., Raviraj M., Sandhar G., Symeonides N., Jones C., Loosemore M., Burgess P.W. (2025). Attentional, Anticipatory and Spatial Cognition Fluctuate throughout the Menstrual Cycle: Potential Implications for Female Sport. Neuropsychologia.

[36] Ronca F., Bruinvels G., Lowery M., Loosemore M., Burgess P.W. (2025). Menstrual Cycle and Athletic Status Interact to Influence Symptoms, Mood, and Cognition in Females. Sports Med. Open.

[37] Colangelo J., Smith A., Buadze A., Liebrenz M. (2023). Ultra Culture–Ultra Reality: A Content Analysis of YouTube Depictions of Ultra Endurance Sport and Comparisons to Scientific Literature. Front. Sports Act. Living.

[38] Smith A., Buadze A., Colangelo J., Liebrenz M. (2023). A Review of Mental Health Issues in High-Performance and Elite-Level Cycling. Int. J. Sports Med..

[39] Chhabra B., Szabo A. (2024). Placebo and Nocebo Effects on Sports and Exercise Performance: A Systematic Literature Review Update. Nutrients.

[40] Broad A., Biswakarma R., Harper J.C. (2022). A Survey of Women’s Experiences of Using Period Tracker Applications: Attitudes, Ovulation Prediction and How the Accuracy of the App in Predicting Period Start Dates Affects Their Feelings and Behaviours. Women’s Health.

[41] Zwingerman R., Chaikof M., Jones C. (2020). A Critical Appraisal of Fertility and Menstrual Tracking Apps for the iPhone. J. Obstet. Gynaecol. Can..

[42] Meijen C., Martin E.A. (2024). ‘I Don’t Want to Be Seen as Period Prone’: An Exploration of Psychological Strategies Used across the Menstrual Cycle. Int. J. Sports Sci. Coach..

[43] Lewis J., Ritchie J. (2003). Generalising from Qualitative Research. Qualitative Research Practice: A Guide for Social Science Students and Researchers.

[44] Berger R. (2015). Now I See It, Now I Don’t: Researcher’s Position and Reflexivity in Qualitative Research. Qual. Res..

[45] Verma S., Kind L.R. (2025). Women’s Intuition as Attunement: A Biological and Social Perspective. Front. Sociol..

